# Application and side effects of blood flow restriction technique: A cross-sectional questionnaire survey of professionals

**DOI:** 10.1097/MD.0000000000025794

**Published:** 2021-05-07

**Authors:** Victor Sabino de Queiros, Matheus Dantas, Gabriel Rodrigues Neto, Luiz Felipe da Silva, Marina Gonçalves Assis, Paulo Francisco Almeida-Neto, Paulo Moreira Silva Dantas, Breno Guilherme de Araújo Tinôco Cabral

**Affiliations:** a Master of Science in Physical Education, Federal University of Rio Grande do Norte (UFRN), Natal, Rio Grande do Norte; b Professional Master's in Family Health/Physical Education Coordination/Physiotherapy Coordination, Faculties of Nursing and Medicine Nova Esperança (FACENE/FAMENE), João Pessoa; c Bachelor of Science in Physical Education, Unifacisa University Center (UNIFACISA), Campina Grande, Paraíba, Brazil.

**Keywords:** KAATSU, physical exercise, rehabilitation, resistance training, side effects, vascular occlusion

## Abstract

The physiological benefits of applying blood flow restriction (BFR) in isolation or in the presence of physical exercise have been widely documented in the scientific literature. Most investigations carried out under controlled laboratory conditions have found the technique to be safe. However, few studies have analyzed the use of the technique in clinical settings.

To analyze how the BFR technique has been applied by professionals working in the clinical area and the prevalence of side effects (SEs) resulting from the use of this technique.

This is a cross-sectional study. A total of 136 Brazilian professionals who perform some function related to physical rehabilitation, sports science, or physical conditioning participated in this study. Participants answered a self-administered online questionnaire consisting of 21 questions related to the professional profile and methodological aspects and SEs of the BFR technique.

Professionals reported applying the BFR technique on individuals from different age groups from youth (≤18 years; 3.5%) to older adults (60–80 years; 30.7%), but mainly on people within the age group of 20 to 29 years (74.6%). A total of 99.1% of the professionals coupled the BFR technique with resistance exercise. Their main goals were muscle hypertrophy and physical rehabilitation. The majority (60.9%) of interviewees reported using BFR in durations of less than 5 minutes and the pressure used was mainly determined through the values of brachial blood pressure and arterial occlusion. Moreover, 92% of professionals declared observing at least 1 SE resulting from the BFR technique. Most professionals observed tingling (71.2%) and delayed onset of muscle soreness (55.8%). Rhabdomyolysis, fainting, and subcutaneous hemorrhaging were reported less frequently (1.9%, 3.8%, and 4.8%, respectively).

Our findings indicate that the prescription of blood flow restriction technique results in minimal serious side effects when it is done in a proper clinical environment and follows the proposed recommendations found in relevant scientific literature.

## 1 Introduction

It has previously been proposed that the minimum intensity of 60% to 70% of 1 repetition maximum (1RM) was necessary for a resistance training (RT) program to improve muscle strength and muscle hypertrophy (MH).^[1,2]^ RT programs consisting of low mechanical load exercise (i.e., 20%–40% of 1RM), associated with blood flow restriction (BFR) caused by the fixation of blood pressure cuffs in the proximal region of the exercised limb, can promote MH^[3,4]^ and increase muscle strength similar to traditional training (i.e., 80% de 1RM).^[3]^ Some mechanisms have been proposed to justify the morphological and structural adaptations provided by the technique, including increased recruitment of high threshold motor units,^[5]^ acute cell swelling,^[6]^ and reduced myostatin gene expression.^[3]^

The possibility of gains in strength and MH related to low mechanical stress makes RT with BFR (RT+BFR) a good alternative for older adults^[7]^ and individuals recovering from orthopedic injuries.^[8,9]^ It becomes valid to add that the adaptations conferred by the application of the BFR are not only imitated to the strength exercises. Previously, it was found that the passive application of BFR (i.e., without exercise) was able to attenuate postoperative atrophy^[10]^ and walking with BFR was able to promote MH of the quadriceps in young men.^[11]^ In addition, this type of exercise seems to maximize antioxidant, metabolic,^[12]^ and cardiorespiratory adaptations.^[11]^

Although the use of the BFR technique can be advantageous in certain contexts, its prescription should be made with caution considering that side effects (SEs) such as rhabdomyolysis,^[13–16]^ retinal vascular occlusion,^[17]^ pulmonary embolism, and thrombus venous^[18]^ can be identified after performing the technique. Even so, these are rare cases and most scientific experiments carried out in laboratories support the safety of BFR technique.^[19–21]^

Previous experimental work regarding BFR provides relevant information to this study. However, it is necessary to carefully analyze the method adopted in each of these scientific works. For example, most studies included in systematic reviews analyzed the effect of RT+BFR on hemodynamics^[20]^ and hemostasis,^[21]^ with an intensity of 20% to 30% at 1RM, short intervals (i.e., 30–60 seconds), and a volume of up to 75 repetitions being adopted. In addition to safety, protocols with these characteristics provide suitable conditions for muscle growth. Therefore, the method was recommended in a document organized by specialists on the subject.^[22]^

The use of BFR in rehabilitation and fitness programs has become popular in various regions of the world^[16]^ and, despite an extensive number of publications on the technique's effectiveness and safety, little is known about how the technique has been applied outside the laboratory and the consequent SEs observed. Research that analyzes this outcome can expand the field of knowledge about technical safety and provide pertinent information for coaches and physiotherapists who are interested in managing the technique. Therefore, the present study aimed to analyze whether the BFR technique has been applied in the practical field according to the recommendations set out in relevant literature, and the prevalence of reported SEs.

## 2 Methods

### 2.1 Subjects

A total of 136 health and fitness professionals working in Brazil participated in the study; however, only 113 reported having prescribed the BFR technique. The descriptive characteristics of the participants are reported in Table 1. The recruitment of participants took place in a nonprobabilistic manner through dissemination on social media. For eligibility criteria, participants were over 18 years old and had performed some activities related to physical rehabilitation, sports science, or physical conditioning. Mandatorily, professionals who were interested in participating in this research should present academic training in Physical Education or Physiotherapy. The research was carried out through an online form composed of 2 parts: the informed consent form and questions related to the use of the BFR technique.

**Table 1:** Characteristics of the professionals who participated in the research.

	Absolute frequency (n = 136)	Relative frequency (%)	Confidence interval (95%)
Academic degree			
Physical education	103	75.3%	68.05%–82.55%
Physiotherapy	33	24.7%	17.45%–31.95%
Gender			
Male	109	80.1%	73.39%–86.81%
Female	27	19.9%	13.19%–26.61%
Age range			
18–29 yr old	67	49.3%	40.90%–57.70%
30–39 yr old	57	41.6%	33.32%–49.88%
40–49 yr old	11	8.1%	3.51%–12.69%
50–59 yr old	1	0.7%	−0.70% to 2.10%
≥ 60 years old	—	—	—
Workplace			
Gym	92	67.6%	59.73%–75.47%
Clinic	26	19.1%	12.49 to 25.71%
Amateur sports club	3	2.2%	–0.27% to 4.67%
Professional sports club	2	1.5%	–0.54% to 3.54%
University	13	9.6%	4.65%–14.55%

### 2.2 Study design

This was a descriptive cross-sectional study carried out between May and June 2020. Recruitment was conducted via social media, and professionals who were interested contacted the researchers working on this study to obtain the online form link. The questionnaire consisted of 21 multiple-choice questions: 5 questions related to the profile of the professionals, 14 questions related to methodological aspects relevant to the application of BFR technique, including age range of people who received the technique, type of application (i.e., passive or combined with physical exercise), duration of restriction, frequency of application, methodology for control restriction pressure, and intensity (% 1RM), volume (number of sets and repetitions) and inter-set recovery interval applied in RT+BFR and its purpose of use (i.e., MH, rehabilitation, physical conditioning, vascular adaptations, heating way), 1 question related to the SEs observed during or after the performance of the technique and 1 question related to contraindications for using the technique. The questionnaire used in our study was developed based on the collection instrument used by Patterson and Brandner^[16]^ and was tested on 3 professionals in the field of physical education who used the technique regularly. This approach has been used previously.^[16]^

### 2.3 Data analysis

Data is presented in descriptive statistics (relative frequency and 95% confidence intervals [CI_95%_]). All frequency analyses were performed using the virtual platform Google Forms, which were also used to develop the questionnaire, and CI_95%_ calculations were performed in Microsoft Excel.

## 3 Ethical considerations

This study is an original contribution which has not been previously published. The research was conducted in accordance with the international guidelines of the Declaration of Helsinki and was approved by the Ethics Committee for Research with Human Beings from the State University of Paraíba, Brazil (protocol no. 4.028.279).

## 4 Results

**Figure 1:**
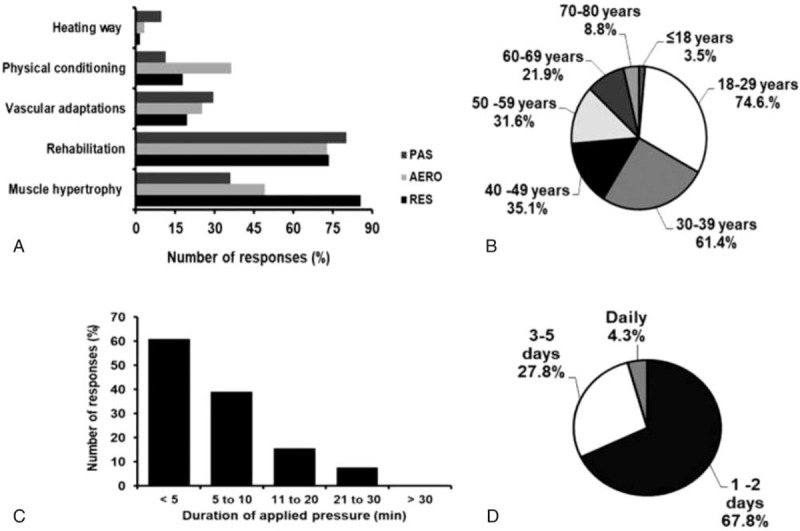
Purpose of use (A), age distribution of people prescribed BFR technique (B), duration of restriction (C) and frequency of use (D). AERO = Aerobic; PAS = Passive; Res = Resisted.

The BFR technique was mainly used in resistance exercises (99.1%; CI_95%_ = 97.36%–100.84%), followed by aerobic exercise (23.5%; CI_95%_ = 15.68%–31.32%), and least of all passive BFR (16.5%; CI_95%_ = 9.66%–23.34%). A greater number of professionals reported individualizing the restriction pressure based on values of brachial blood pressure (BP; 44.6%; CI_95%_ = 35.43%–53.77%), followed by relative values of the arterial occlusion pressure (AOP; 38.4%; CI_95%_ = 29.39%–47.37%), and least of all subjective perception of discomfort (17%; CI_95%_ = 10.07%–23.93%). The age distribution of subjects prescribed BFR technique, duration of restriction, purpose of prescription, and frequency of application are reported in Figure 1.

### 4.1 Resistance exercise plus BFR

**Figure 2:**
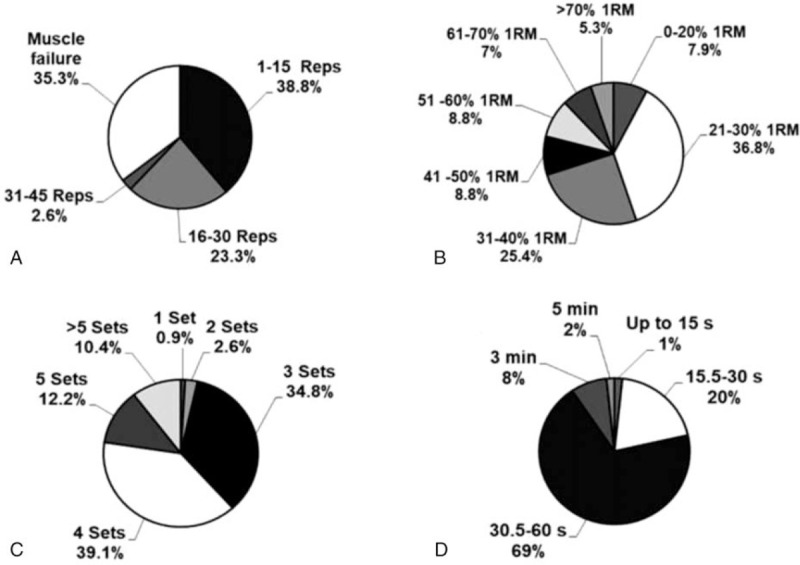
Number of repetitions (A), intensity (B), number of sets (C), and recovery interval between sets (D) used in RT + BFR. min = minute, Reps = repetitions, RM = repetition maximum, RT+BFR = resistance training with blood flow restriction, s = seconds.

Most professionals reported (36.8%; CI_95%_ = 27.91–45.69) that they were prescribed BFR training with intensities of 21% to 30% of 1RM, followed by 31% to 40% 1RM (25.4%; CI_95%_ = 17.37%–33.43%), 41% to 50% 1RM (8.8%; CI_95%_ = 3.58%–14.02%), and 51% to 60% 1RM (8.8%; CI_95%_ = 3.58%–14.02%). The main repetition volume used was 1 to 15 (38.8%; CI_95%_ = 29.82%–47.78%), followed by training to muscle failure (35.3%; CI_95%_ = 26.49%–44.11%), 16 to 30 repetitions (23.3%; CI_95%_ = 15.51%–31.9%), and finally 31 to 45 repetitions (2.6%; CI_95%_ = 0.33%–5.53%). Regarding set volume, 39.1% (CI_95%_ = 30.10%–48.10%), 34.8% (CI_95%_ = 26.02%–43.58%), and 12.2% (CI_95%_ = 6.17%–18.23%) of professionals reported employing 4, 3, and 5 sets, respectively. A total of 69% (CI_95%_ = 60.47–77.53%) of professionals selected recovery intervals between sets of 30.5 and 60 seconds, while intervals between 15.5 and 30 seconds and 3 minutes were selected by 20% (CI_95%_ = 12.62%–27.38%) and 8% (CI_95%_ = 3.00%–13.00%) of the interviewed professionals, respectively. The data are presented in percentages in Figure 2.

### 4.2 Side effects and contraindications

**Figure 3:**
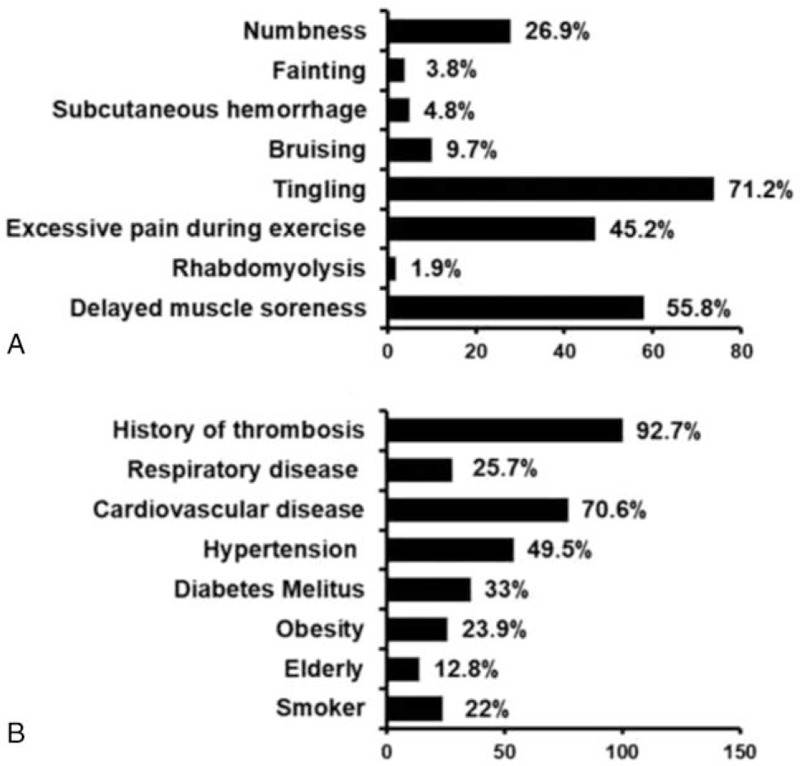
Reported side effects (A) and contraindications (B) for using BFR technique.

Regarding SEs, it was found that 104 (92%) of the professionals reported having already identified an effect during or after the BFR technique. The main SEs observed were tingling (71.2%), followed by delayed onset muscle soreness (DOMS) (55.8%) and excessive pain during exercise (45.2%). Rhabdomyolysis, fainting, and subcutaneous hemorrhaging were reported less frequently (1.9%, 3.8%, and 4.8%, respectively). The main contraindications for the use of the technique were a history of thrombosis (92.7%) and cardiovascular disorder (70.6%). The main SEs and contraindications for using the technique are shown in Figure 3.

## 5 Discussion

This study analyzed how the BFR technique is applied by professionals who work in clinical settings and the prevalence of SEs resulting from the technique. Thus, our main findings were: Most professionals reported using BFR in RT; MH and rehabilitation were the main goals pursued by the professionals who prescribed the BFR technique; The individualization of restriction pressure mainly occurred through the brachial BP and AOP values; Regarding SEs, tingling and DOMS were most frequently reported; Subcutaneous hemorrhaging, fainting, and rhabdomyolysis were reported less frequently.

Our data showed that the main goals of using the BFR technique were MH and rehabilitation. Several studies over the past 20 years have found significant increases in muscle strength and MH after low-load physical training programs associated with BFR.^[3,4,23]^ In addition, passive BFR was able to alleviate postoperation atrophy due to disuse.^[10]^ Therefore, training with BFR is effective for fitness and rehabilitation, and MH and physical rehabilitation were the main outcomes sought by professionals in the field who used the technique.

The BFR technique was mainly applied in resistance exercises (99.1%) for periods shorter than 10 minutes. Most of the interviewed professionals prescribed the RT+BFR variables according to the recommendations proposed in the relevant literature (i.e., 4 sets, interspersed with recovery periods of 30–60 seconds, between 20% and 40% of 1RM).^[22]^ Training based on these recommendations can promote a 9% and 6.6% increase in the cross-sectional area of the triceps brachial^[23]^ and quadriceps,^[7]^ respectively. A small portion (5.3%) of professionals reported using BFR in high-intensity exercises. It was previously verified that the application of BFR in a high-intensity-RT program was not able to promote additional benefits in quadriceps strength and hypertrophy.^[24]^

Less than half (38.4%) of the professionals indicated individualizing the restriction pressure based on the values of the exercised AOP, which is a method developed by Laurentino et al ^[3]^ and is considered the gold standard for prescribing training pressure. Furthermore, 44.6% of professionals use brachial BP values to relativize the restriction pressure. This measure was previously used to individualize the pressure levels used in exercises with BFR.^[25–28]^ However, this method was criticized for disregarding the size of the cuff used and the anthropometric characteristics of the limb.^[29,30]^ For example, 130% of the brachial systolic BP is the most frequently used protocol in scientific studies, and was sufficient to generate limb arterial occlusion in 49 out of 116 individuals when the restriction was performed with a 13.5 cm cuff. On the other hand, the use of the same external pressure with a 5 cm cuff was only able to generate total limb arterial occlusion in 1 out of 86 subjects.^[30]^

Since BFR training has the goal of preventing venous return without blocking arterial flow, a restriction based on AOP values may be more appropriate. Patterson and Brandner^[16]^ found that only 11.5% of 115 professionals reported using relative values of AOP to establish the restriction pressure. The number of professionals in the current and the aforementioned study who prescribed pressure based on AOP values can be justified by the period in which each study was conducted. The increase in the number of scientific studies that employed the procedure^[4,31,32]^ and the development of predictive equations with the capacity to estimate occlusion values of a limb^[33,34]^ may have contributed to the increase in the number of professionals who use AOP values when prescribing the BFR technique.

The BFR technique is already used worldwide in clinical settings.^[16]^ Therefore, studies that analyze possible complications arising from the use of BFR are currently relevant. We found that some professionals in our study identified tingling and numbness during exercise with BFR. This type of response has been previously observed.^[18,35]^ Although it disappeared after pressure release, the numbness observed was related to a possible nerve conduction blockage.^[19]^ There was no chronic effect on nerve conduction associated with 4 weeks of RT+BFR,^[36]^ which demonstrates the relative safety of RT+BFR.

DOMS was also reported by the interviewed professionals. The presence of DOMS after exercises with BFR has been observed in previous studies that evaluated the effect of the BFR technique on exercise-induced muscle damage.^[37–39]^ However, it should not be a cause for concern, considering that this SE mainly affected individuals who were not used to physical exercise and disappeared in a few days, dissipating as the individual continued to exercise.^[37]^ Nevertheless, the risk of excessive rhabdomyolysis (i.e., exacerbated muscle damage) characterized by excessive leakage of muscle proteins which can generate renal failure cannot be dismissed.^[40]^

Although few cases were observed after exercise with BFR in this and previous studies,^[16,18]^ the possibility of rhabdomyolysis should not be neglected and certain precautions must be taken. For example, the use of sets to volitional failure on RT+BFR seems to be an aggravating variable for exercise-induced muscle damage.^[37]^ Adaptations promoted by can occur without reaching muscle failure.^[41]^ Therefore, this strategy could be avoided, especially in clinical contexts.

Some of the interviewed professionals reported cases of subcutaneous hemorrhaging and fainting resulting from exercise with BFR. The subcutaneous hemorrhaging was transient and resolved quickly, even if the training session continued.^[18]^ Regarding fainting, Nakajima et al ^[18]^ point out that this response is justified by a reduction in cardiac preload due to the reduction in venous return generated by the application of BFR in the thigh. Reducing the preload decreases cardiac output and consequently blood flow to the brain. Martín-Hernández et al ^[42]^ add that the exacerbated vasodilator effect promoted by the BFR technique through shear stress can induce hypotensive syncope.

Cardiovascular disorders, hypertension, and a history of thrombosis were the main concerns of professionals regarding BFR technique users. Stasis (i.e., impaired blood flow) is one of the risk factors for thrombogenesis.^[43]^ Thus, exercise duration and the pressure levels applied in BFR training seem insufficient for thrombi formation.^[44]^ In a sample of 12,642 people who underwent the BFR technique, there were 7 cases (0.055%) of venous thrombosis and 1 case (0.008%) of pulmonary embolism.^[15]^ Also, experimental studies have not been successful in demonstrating any negative effect of BFR training on hemostasis.^[36,45]^

Regarding hemodynamic aspects, Spranger et al^[46]^ draw attention to possible undesirable responses when performing BFR exercises. The authors state that the metabolic stress induced by the attenuation of blood flow can exacerbate hemodynamic responses via increased activity of the exercise pressor reflex. A meta-analysis that supports this statement found that associated low-load RT+BFR promotes higher BP values when compared with the traditional RT performed with an intensity of ≥60% of 1RM or < 60% of 1RM.^[47]^ However, this does not mean that hypertensive and cardiac patients cannot perform exercises with BFR; there are some ways to attenuate the magnitude of hemodynamic responses during this type of exercise, such as using intermittent BFR.^[20]^

The present study has some limitations that warrant highlighting: The data are limited to the use of the blood flow restriction technique by professionals exclusively working in Brazil; This study has a limited number of questions about the use of the technique passively or in aerobic exercise; Data collection was performed using an online form.

## 6 Conclusion

The present study provided an overview of the clinical use of blood flow restriction technique. The results showed that the recommendations imposed in scientific literature have mostly been applied in practical fields. This aspect is of particular interest, as it provides evidence that professionals who are in clinical environments (gyms and physiotherapy clinics) consider safety when prescribing exercises using the blood flow restriction technique. In addition, we found that a low number of professionals showed serious side effects such as rabidomyolysis, syncope, and subcutaneous hemorrhage.

## Acknowledgments

The authors are grateful to the Coordination for the Improvement of Higher Education Personnel (CAPES) for granting financial support to researchers VSQ, MD, and PFAN.

## Author contributions

**Conceptualization:** Victor Sabino de Queiros e Breno Guilherme de Araújo Tinôco Cabral.

**Data curation:** Victor Sabino de Queiros, Matheus Dantas, Luiz Felipe da Silva.

**Methodology:** Victor Sabino de Queiros, Matheus Dantas, Gabriel Rodrigues Neto, Breno Guilherme de Araújo Tinôco Cabral.

**Supervision:** Breno Guilherme de Araújo Tinôco Cabral e Paulo Moreira Silva Dantas.

**Writing – original draft:** Victor Sabino de Queiros, Matheus Dantas, Gabriel Rodrigues Neto, Luiz Felipe da Silva, Marina Gonçalves Assis, Paulo Francisco Almeida Neto.

**Writing – review & editing:** Victor Sabino de Queiros, Matheus Dantas, Gabriel Rodrigues Neto, Luiz Felipe da Silva, Marina Gonçalves Assis, Paulo Francisco Almeida-Neto, Paulo Moreira Silva Dantas, Breno Guilherme de Araújo Tinôco Cabral.
